# Auto-collimation diffraction of two-dimensional metal–dielectric grating with azimuth angle of 45°

**DOI:** 10.1515/nanoph-2024-0399

**Published:** 2025-01-03

**Authors:** Yi-Han Wang, Jin Wang, Yu-Da Chen, Zhi-Sen Huang, Wei Jia, Chang-He Zhou

**Affiliations:** College of Physics & Optoelectronic Engineering, 47885 Jinan University, Guangzhou, Guangdong 510632, China

**Keywords:** two-dimensional grating, polarization independence, high efficiency

## Abstract

Grating under auto-collimation configuration with polarization-independent high diffraction efficiency plays an important role in the displacement measurement system, spectral beam combining system and so on. In this paper, we proposed, for the first time, a reflective two-dimensional metal-dielectric grating of which the (−1, −1) order beam is diffracted back along the input light direction, when the incident azimuth angle is 45°. With optimized structure, the (−1, −1) order diffraction efficiencies of transverse electric polarization (TE) and transverse magnetic polarization (TM) are 95.01 % and 95.04 % at incident wavelength of 632 nm, respectively. The structure based on the frustum of a cone performs well in manufacturing tolerance, which provides possibility for practical applications. A grating is fabricated experimentally in this research. The high efficiencies of TE and TM polarization have great application potential in 2D displacement measurement technique and high power laser systems.

## 1 Introduction

Auto-collimating structure, also known as Littrow mounting, is a special configuration of grating diffraction in which the diffracted light returns in the direction of the incident light. They are commonly used in spectral interferometers [1], [2], pulse compression [3], [4], spectrometers [5], [6], and beam combining system [7], [8], [9]. In 2016, Lu et al. presented a grating interferometer based on one-dimensional grating in auto-collimation configuration [9]. In 2024, Zhou et al. proposed bidirectional a Littrow double grating interferometry system, which provides a quadruple optical subdivision in the case of single-diffraction [10]. In 2013, Vulcan 10 PW project designers also chose auto-collimating gratings in Littrow configuration for their compressors [11]. In 2013, Bai et al. presented a multi-pulse picosecond laser with grating stretching and compression technologies [12]. In 2011, Gomer et al. used two one-dimensional gratings in Littrow configuration to build an ultraviolet (UV) Raman spectrometer suitable for space planetary missions [13]. In 2022, Németh et al. proposed a tunable spatial heterodyne spectrometer using an auto-collimating grating. The single-grating design makes it very easy to tune the spectral range to the desired spectral region [14]. In 2001, Hawthorn et al. have developed an enhanced Littrow configuration extended cavity diode laser that can be tuned without changing the direction of the output beam [15]. In 2017, Kappa et al. numerically analyzed the electromagnetic behavior of a dynamically reconfigurable spatial terahertz wave modulator based on a micromirror grating in Littrow configuration [16]. In 2024, Li et al. proposed a novel flat-field, dual-optic imaging EUV – soft X-ray spectrometer. The systems use a grating with blaze angle and azimuth to achieve aberration-corrected first-order beam spots [17]. In 2020, Xie et al. proposed that transmission grating are more than 95 % with incident wavelength ranging from 1,446 to 1,641 nm and incident angle ranging from 32.1 to 42.7° for both TE and TM polarizations in Littrow configuration [18].

With the increasing application of 2D gratings [19], [20], [21], the auto-collimating structure of 2D gratings has also been widely investigated. In 2022, Yin et al. proposed a 3D measurement method based on two-dimensional grating with Littrow equal-optical path incidence to detect the 3D displacement in *X*, *Y* and *Z* directions [22]. In 2018, Chen et al. proposed a polarization-independent two-dimensional metallic dielectric grating, of which the efficiencies at TE and TM polarization were 74.8 % and 68.2 % [23]. In 2020, Zhou et al. proposed a two-dimensional grating based on column-hole nano-arrays for interferometer, which can achieve an ultra-high diffraction efficiency of 98 % at the (−1, 0) order [24], but it is difficult to etch Ta_2_O_5_ with cylindrical hole structure. In 2024, Huang et al. proposed a hovel cylinder array two-dimensional grating, whose efficiencies of (−1, 0) order for p-polarization and s-polarization each 98.54 % and 98.24 % [25]. In 2023, Dong et al. experimentally demonstrate retroreflections with unpolarized absolute efficiency higher than 98 % (99 % in design) at 1,030–1,090 nm using multilayer freeform metagratings [26]. However, due to the complexity of the structure, the metagratings may not be suitable for large-scale manufacturing [27], [28].

However, two-dimensional grating is usually used in the auto-collimating configuration of azimuth angle 0°. For instance, in the photolithography positioning system, four 2D gratings and encoders are distributed around the perimeter of the chuck, and the grating period direction is at an angle of 45° to the edge, which implies a more sophisticated fabrication process [29]. In this study, we proposed a polarization-independent metal dielectric grating with azimuth angle of 45°. The diffraction efficiencies of (−1, −1) orders under TE and TM polarization are 95.04 % and 95.01 % under Littrow-mounting, respectively. In addition, the diffraction characteristics under different incident wavelengths and incident angles are given, the manufacturing tolerances are analyzed and the feasibility of its fabrication are revealed, showing that this grating is simple to make and has acceptable performance. The high diffraction efficiency and polarization-independent characteristics make the grating a promising competitor in the application of the displacement measurement system.

## 2 Structure and materials

As an optical element, diffraction grating can control the propagation direction and power distribution of light by controlling its parameters. The two-dimensional grating equations are,

(1) $$\begin{array}{c} \sin\theta_{m,n}\sin\varphi_{m,n}=\sin\theta \cos\varphi +m\lambda /\Lambda_{y}\\ \sin\theta_{m,n}\cos\varphi_{m,n}=\sin\theta \cos\varphi +m\lambda /\Lambda_{x} \end{array}$$

where *φ* and *θ* are the azimuth and incident angle of the incident light, *φ*_*m*,*n*_ and *θ*_*m*,*n*_ represent the exit azimuth angle and diffraction angle of (*m*, *n*)th order diffracted light. Λ_*x*_ and Λ_*y*_ represent the periods in *x* and *y* directions, which are set to be the same value as Λ in this paper. The working wavelength of the grating is *λ* = 632 nm. When the azimuth angle *φ* = 45°, to meet auto-collimation condition, the incident angle should be the same as the diffraction angle of (−1, −1) order, i.e.,

(2) $$\theta =\theta_{\left(-1,-1\right)}=\text{a}\sin\left(\lambda /\sqrt{2}\Lambda \right)$$

In order to direct most of the light wave energy into the (−1, −1) order, we can choose the appropriate wavelength and grating period, so that only (0, 0), (−1, 0), (0, −1) and (−1, −1) diffraction orders exist, and high diffraction orders become evanescent wave. In this case, the period satisfies,

(3) $$\sqrt{2}\lambda /2<\Lambda <\sqrt{2}\lambda$$

**Figure 1:**
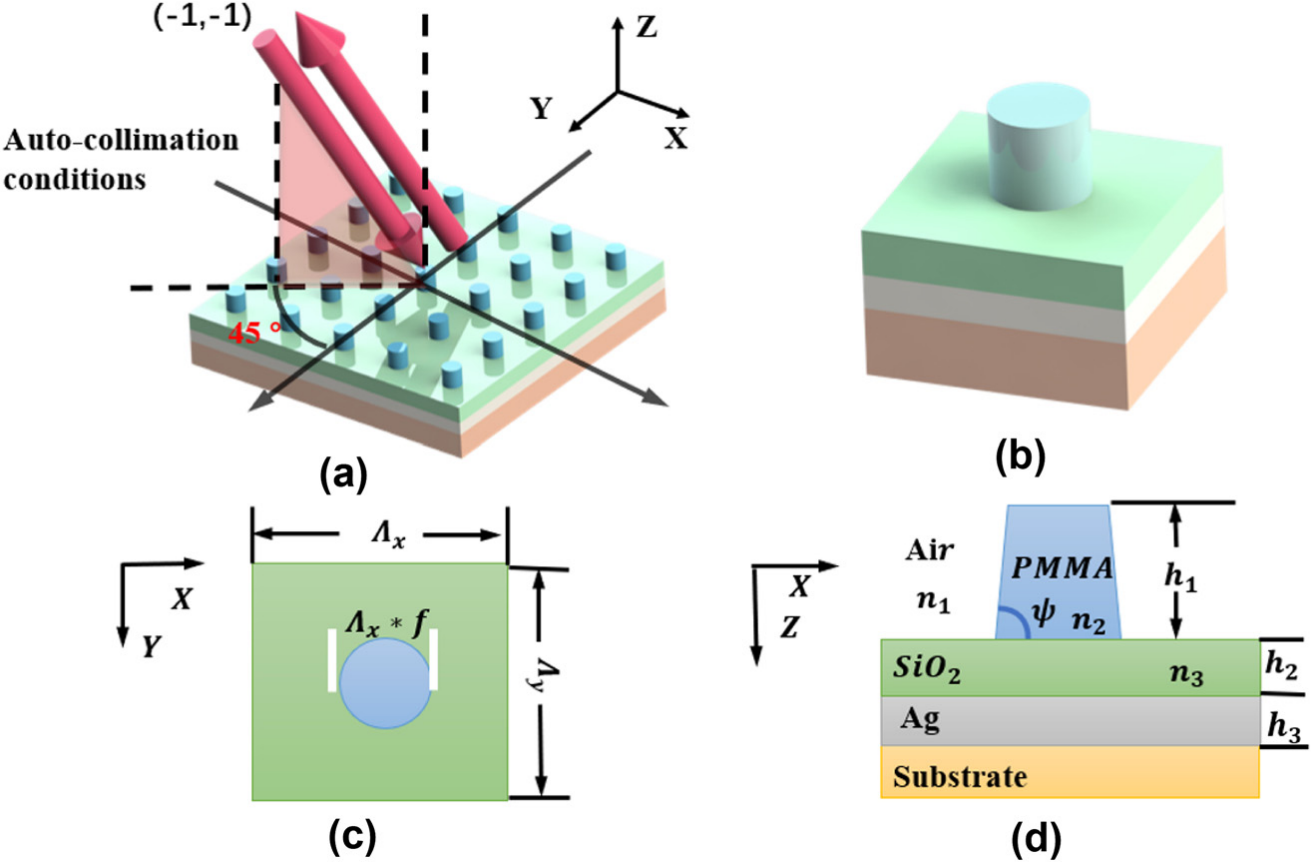
Diagram of grating. (a) The proposed 2D grating structure; (b) unit-cell of the proposed structure; (c) unit-cell seen from top view (*X*–*Y* plane); (d) unit-cell seen from side view considering sidewall angle *ψ* (*X*–*Z* plane).

The structure of the grating from bottom to top is shown in Figure 1. The substrate of the grating is Corning’s 7980 series glass. This is mainly because this glass supports low coefficient of thermal expansion for a wide range of temperatures, and the glass series is complete, which is conducive to the measurement of the film layer of the grating during the experiment. The second layer is a high reflective layer, and the main material is Ag. This is mainly based on the fact that silver has a very high reflectivity in the visible light region. The third layer is phase modulation layer of silicon dioxide, with a height of *h*_2_. The structure of the grating layer on the top layer is cylindrical, with a height of *h*_1_ and a sidewall angle of *ψ*. The main material of the grating layer is polymethyl methacrylate (PMMA). This is because PMMA is can reduce the manufacturing process and can be obtained without etching. Moreover, it is structurally stable after exposure and in lower cost. The refractive indices of Ag, SiO_2_ and PMMA film at the operating wavelength of 632 nm are 0.15107 + *i*4.1180, 1.4571 and 1.4887, which are taken from Refs. [30], [31], [32].

## 3 Parameter optimizations

Based on the symmetry characteristics of the grating, under the azimuth angle of 45°, the diffraction efficiency of the (−1, 0) order of the grating is consistent with the diffraction efficiency of the (0, −1) order. The proposed grating is a periodic structure with multiple materials. Since the structure will affect the diffraction efficiency and bandwidth of the grating, multiple parameters need to be optimized in the design process, to achieve polarization-independent high diffraction efficiency in (−1, −1) order. Therefore, during the design process, the period Λ, cylinder height *h*_1_, the sidewall angle *ψ*, thickness of silica layer *h*_2_ and the duty cycle *f* (The ratio of the diameter of the cylinder to the period of the grating in the *X*–*Y* direction) have significant influences on the diffraction efficiency.

During designing, the thickness of the silver layer *h*_3_ is set to 100 nm, to ensure almost all the light in the visible light band is reflected. In order to obtain accurate numerical solution, we use the rigorous coupled wave analysis (RCWA) and simulated annealing (SA) algorithm to calculate and optimize the diffraction behavior. RCWA is a method that expands the electromagnetic field in the phase modulation field according to diffraction orders, and determines the amplitude of each order by solving the coupled wave differential equations in the phase modulation area [33], [34]. The SA uses the metropolis criterion and appropriately controls the temperature drop process to quickly solve the global optimization problem. The cost function (CF) is defined as $\text{CF}=2-\left[\eta_{\left(-1,-1\right)\text{TE}}^{2}+\eta_{\left(-1,-1\right)\text{TM}}^{2}\right]$ , where $\eta_{\left(-1,-1\right)\text{TE}}$ and $\eta_{\left(-1,-1\right)\text{TM}}$ represent the diffraction efficiency of (−1, −1) order under TE and TM polarizations. When $\eta_{\left(-1,-1\right)\text{TE}}$ and $\eta_{\left(-1,-1\right)\text{TM}}$ reach the maximum diffraction efficiency, the cost function is the lowest and the optimal solution is obtained. The optimized grating parameters are: Λ = 852 nm, *h*_1_ = 768 nm, *h*_2_ = 268 nm, *f* = 0.65. At this time, the diffraction efficiency $\eta_{\left(-1,-1\right)\text{TE}}$ = 95.01 %, $\eta_{\left(-1,-1\right)\text{TM}}$ = 95.04 %, almost all the light energy is concentrated in the (−1, −1) order.

**Figure 2:**
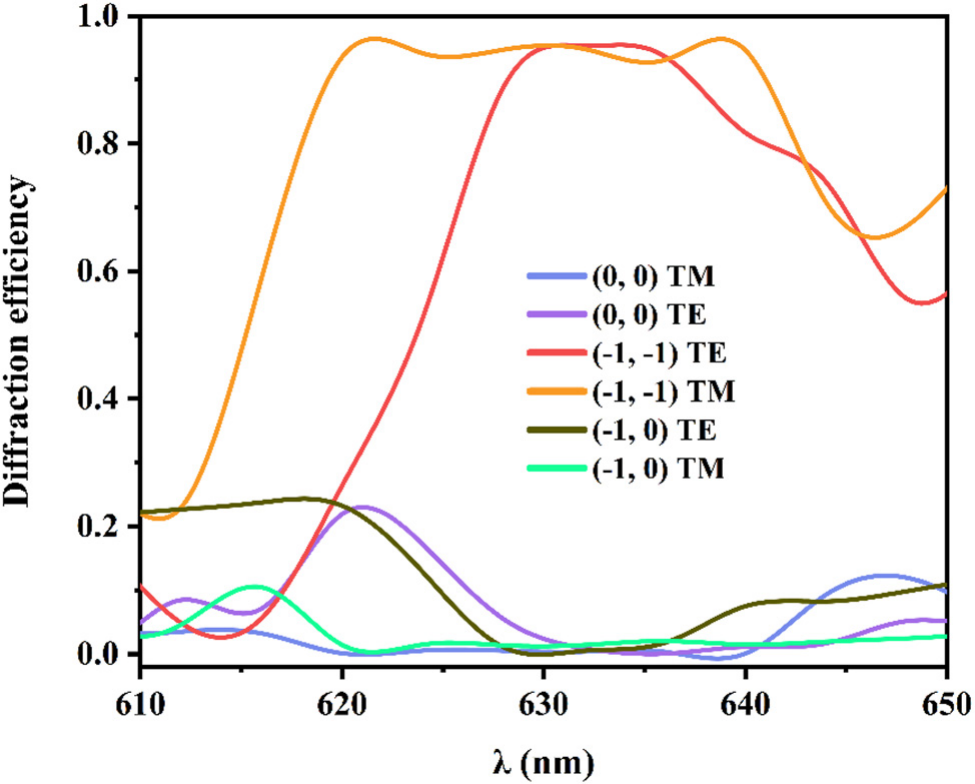
Diffraction efficiency versus wavelength with the optimized grating parameters.

**Figure 3:**
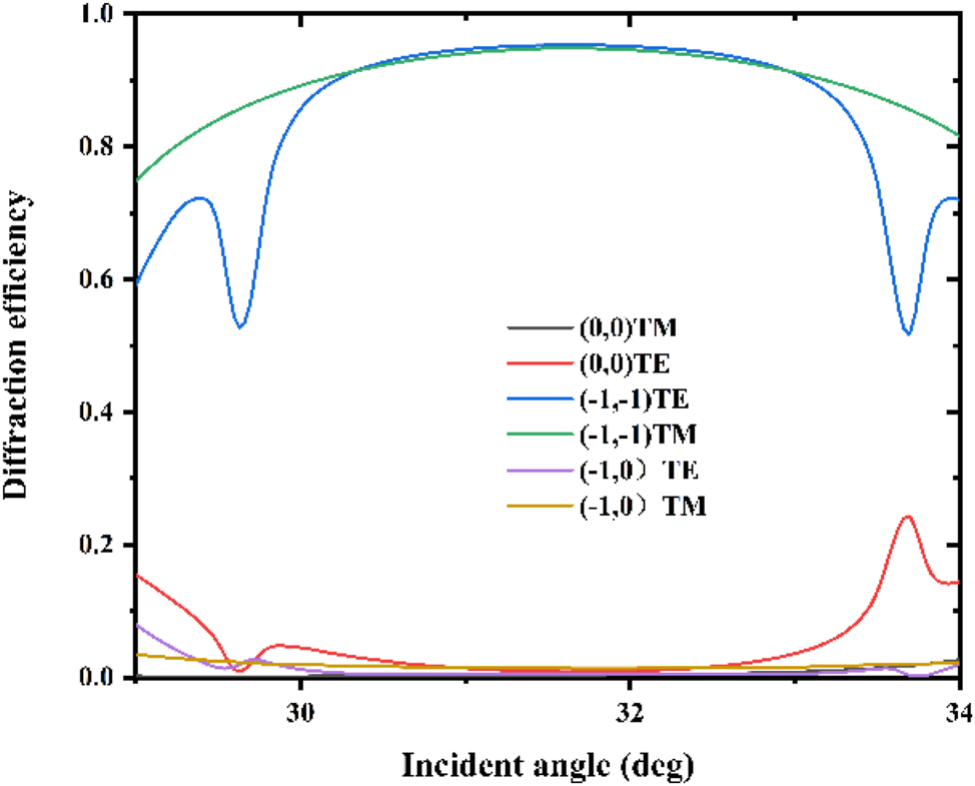
Diffraction efficiency versus incident angle for a wavelength of 632 nm with the optimized grating parameters.

**Figure 4:**
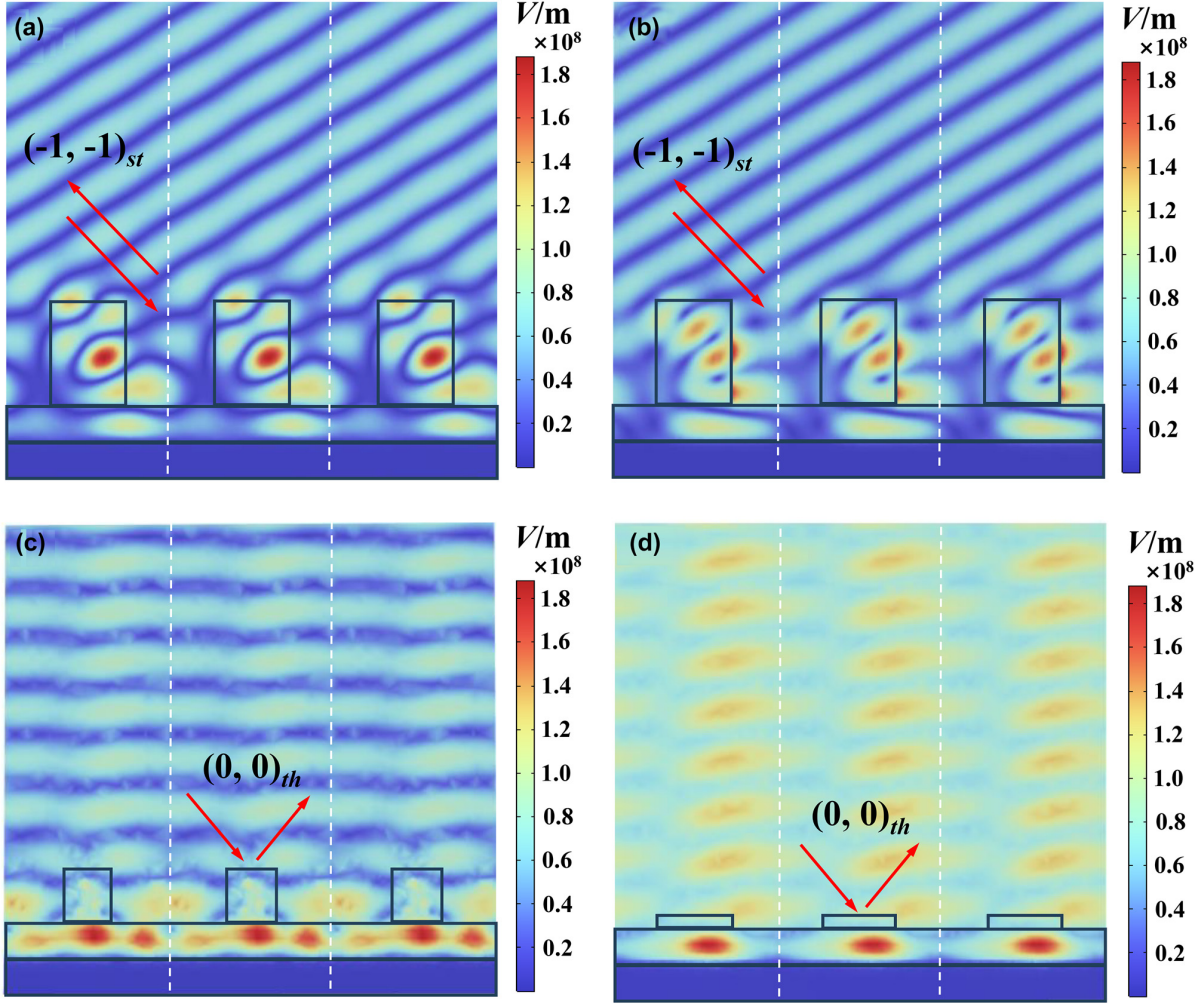
The electric field distributions of the grating with optimized structure for (a) TE polarization and (b) TM polarization, and the electric field distributions of the grating without optimization for (c) TE polarization and (d) TM polarization.

In the optimal solution, the grating has extremely high diffraction efficiency and is polarization independent. However, the grating faces installation errors and wavelength drift caused by long-term operation of the laser. Therefore, it is necessary to analyze the impact of the incident wavelength and incident angle on the grating diffraction efficiency. Figure 2 shows that when the grating is in the optimal solution, the diffraction efficiency changes with the incident wavelength. When the wavelength is far away from 632 nm, the diffraction efficiency of the grating decreases slightly. The optimized grating has an efficiency of more than 90 % at the wavelength ranging from 627 nm to 636 nm. Figure 3 shows the variation of diffraction efficiency with the incident angle. Among them, the diffraction efficiency of the (−1, −1) order does not change too much. The optimized grating has an efficiency of more than 90 % at the incident angle ranging from 30.2° to 32.5°, which meets the practical requirement. Figure 4(a) and (b) show the electric field distributions for TE and TM polarizations of the designed grating calculated by the FEM [35]. When light at 632 nm impinges the grating under Littrow mounting, most of the electric flied energy is diffracted into (−1, −1) order, which forms fringes perpendicular to the diffraction direction. Figure 4(c) and (d) show the electric field distributions when the grating is not optimized, the electric field energy is reflected like a mirror, with minimal observable diffraction phenomenon. This indicates that the designed grating structure minimizing the efficiency of the (0, 0) order, while maximizing the diffraction efficiency of the (−1, −1) order.

## 4 Tolerance analysis

**Figure 5:**
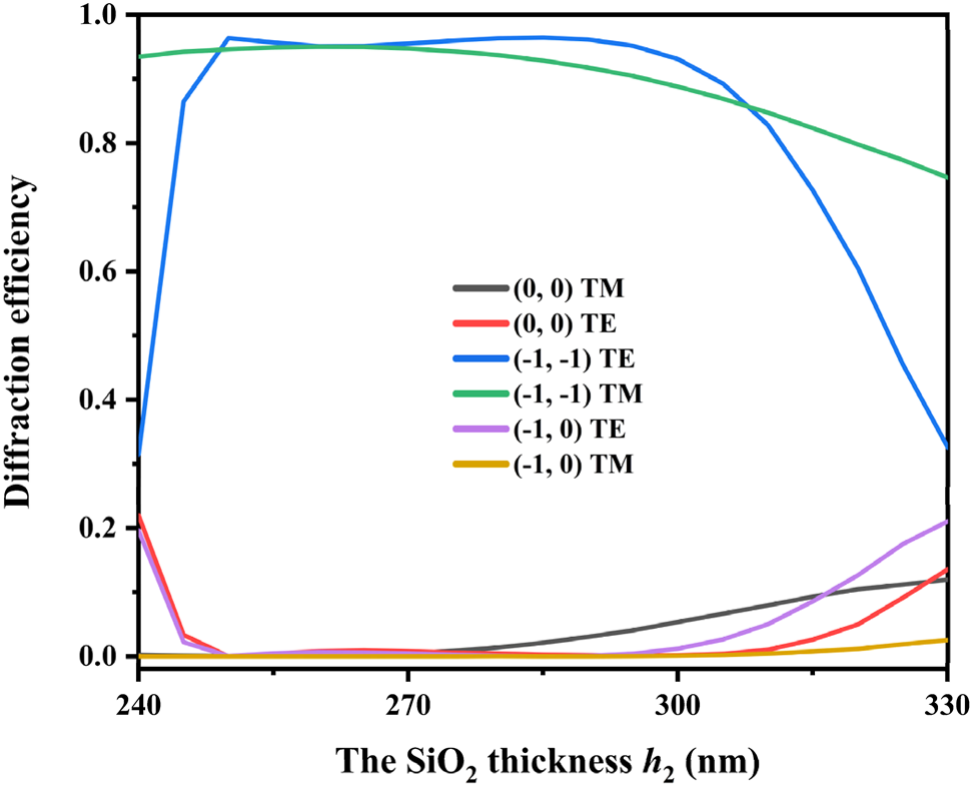
Diffraction efficiency versus SiO_2_ thickness for a wavelength of 632 nm with the optimized grating parameters.

When it comes to the grating manufacturing, the structure often deviates from the designed parameters. Therefore, in order to optimize the manufacturing process and reduce the impact of manufacturing errors on grating performance, it is necessary to analyze the tolerance of the grating structure. The manufacturing of gratings includes 5 steps, namely metal coating, chemical deposition, coating, exposure and development. Figure 5 shows the effect of the thickness of silica (*h*_2_) on the diffraction efficiency. It can be seen from the figure that when *h*_2_ ranges from 245 nm to 295 nm, the diffraction efficiency is above 90 %. There is a manufacturing tolerance of over 50 nm which is enough to ignore the influence of deposition error.

**Figure 6:**
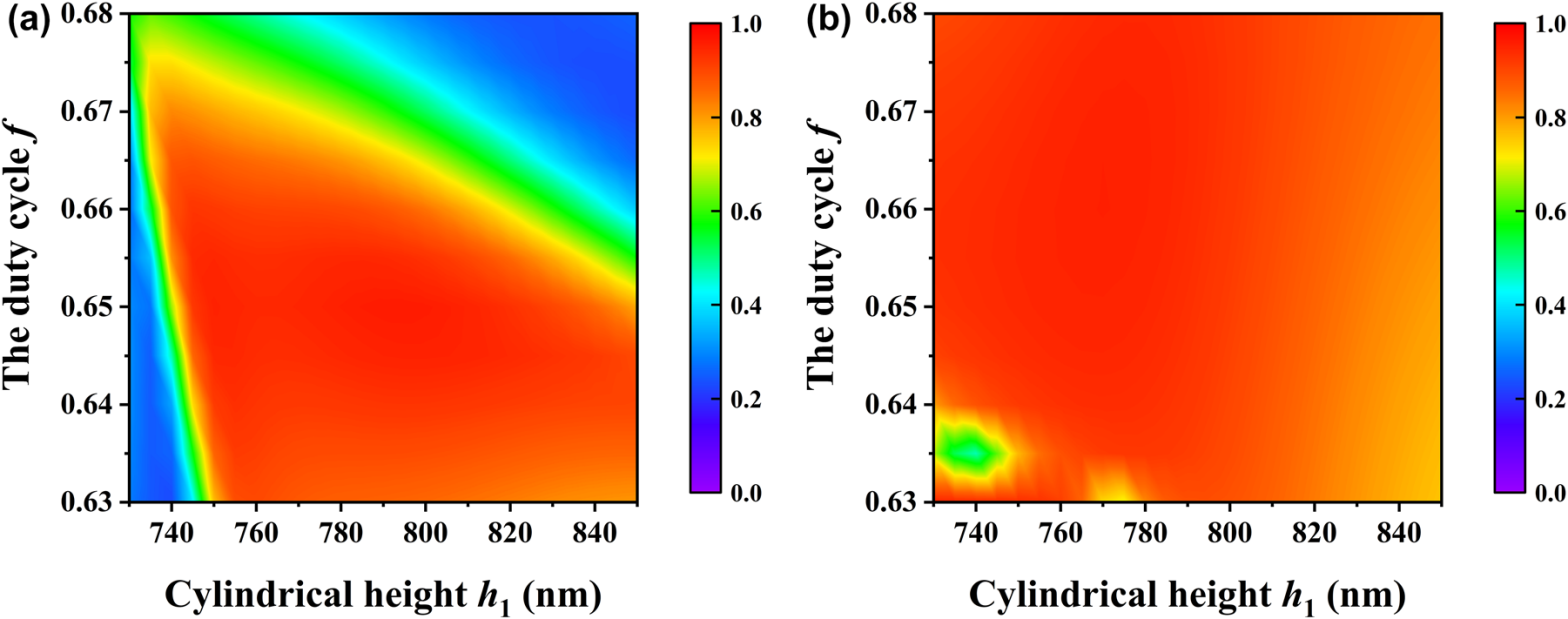
Diffraction efficiency versus cylindrical height and the duty cycle when the sidewall angle is 90° for (a) TE polarization and (b) TM polarization.

In addition to the thickness of the film layer, when the grating is exposed, the morphology of the grating surface is often different from the expected one. Therefore, it is necessary to analyze the morphology and size of the grating surface. The surface structure is cylindrical, which is mainly influenced by the diameter and height of the cylinder and cylindrical sidewall during exposure. Figure 6 shows diffraction efficiency versus cylindrical height and the duty cycle when the sidewall angle is 90°. The 90 % diffraction efficiency can be found when the cylindrical height ranges from 750 nm to 800 nm and the duty cycle is 0.63–0.66. This area shows that the grating has a large manufacturing tolerance at the height of the cylinder, but needs finer control over the diameter. Although, the tolerance under TE polarization is not as large as that under TM polarization, there is still a good manufacturing tolerance, allowing the grating to achieve both high efficiency and polarization independent diffraction.

**Figure 7:**
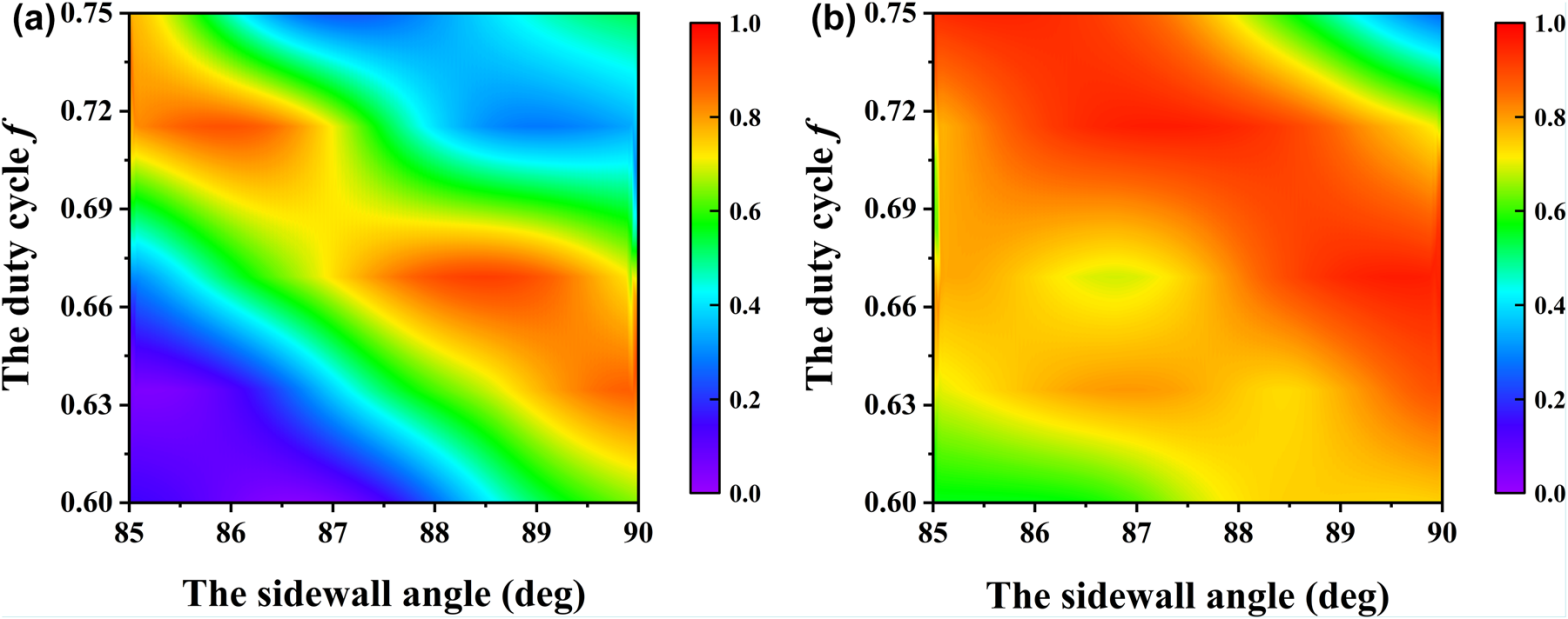
Diffraction efficiency versus the sidewall angle and the duty cycle when the cylindrical height is 778 nm for (a) TE polarization and (b) TM polarization.

Figure 7 shows diffraction efficiency versus the sidewall angle and the duty cycle when the cylindrical height is 778 nm. When the sidewall angle is 88–90° and the duty cycle is 0.63–0.66, the diffraction efficiency is above 90 %. The figures show that when the sidewall angle reduces, the duty cycle should increase to ensure high diffraction efficiency. In order to reduce the difficulty of grating manufacturing, the cylinder should be made as steep as possible. When the sidewall angle of grating is 90°, there is a manufacturing tolerance of 50 nm in height and a tolerance of nearly 20 nm in diameter.

## 5 Experiment and discussion

**Figure 8:**
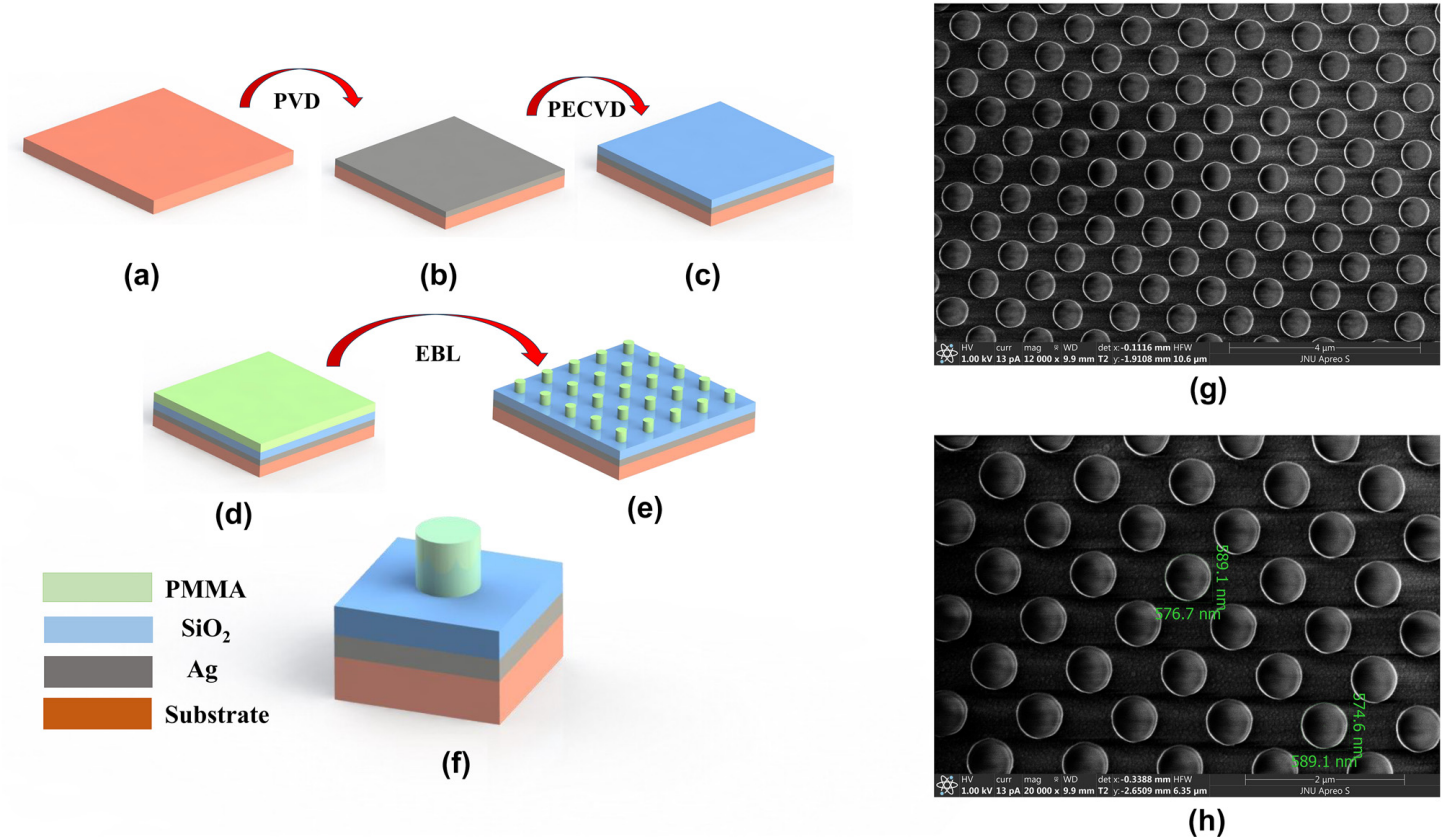
The manufacturing process of the grating; (a) substrate; (b) electron beam evaporation silver plating; (c) plasma enhanced chemical vapor deposition SiO_2_; (d) surface spin-coated photoresists; (e) resulting 2D periodic surface mask after electron beam exposure and development; (f) unit-cell; (g)–(h) SEM micrographs of grating in photoresist generated by use of electron-beam writing.

In order to verify calculation results, we fabricated the grating based on electron beam direct writing technique. The experimental process is divided into 5 steps, as shown in Figure 8(a)–(f). Firstly, a 100 nm silver layer is deposited on the substrate as a high-reflective layer. Secondly, Vapor chemical deposition was used to deposit 268 nm silicon dioxide on the surface of the silver layer at a deposition rate of 71 nm/min. After interference measurement with an ellipsometer, the refractive index of the layer is 1.46 and the thickness is 266 ± 5 nm. The third step is to spin-coat 780 nm photoresist (polymethyl methacrylate (950 k, MicroChem, 11 % in anisole (A7)) on the surface of the silicon dioxide layer (d). Then by using the electron beam direct writing lithography system (Raith. B.V/EBPG5150), with an acceleration voltage of 50 kv and a beam current of 5 nA, 2D periodic structures in an area of 3 mm × 4 mm are patterned. Last, after development of the PMMA, the desired cylinder grating structure is obtained. In the ideal condition, the grating sidewall is steep and straight, but in the fabrication process the grating sidewall angle tends to be smaller than 90°. We show the sidewall angle of the grating in Figure 1(d).

The micrograph of the grating obtained by a scanning electron microscope (SEM) is shown in Figure 8. We can see from Figure 8(g) and (h) that the grating period is around 850 nm, and the grating cylinder diameter is about 576 nm. According to this scale, the duty cycle of the grating is nearly 0.67, which will affect the diffraction efficiency of the grating.

In order to evaluate the diffraction characteristics of the grating, the beam of the red laser (HNL210LB, Thorlabs) was emitted to the grating surface under auto-collimation configuration, after passing through polarizing beam splitter (PBS), and the diffraction efficiency was obtained by measuring the intensity of the diffracted light at each order. Table 1 shows the measured diffraction efficiency and theoretical diffraction efficiency when the incident wavelength is 632 nm with the fabrication parameter. And the third column of the results is the efficiency of the optimized parameters. The diffraction efficiency of the (−1, −1) order of this grating under TE and TM polarization is 76.5 % and 59.64 %. In addition, the simulation results of the measured parameters are basically consistent with the experimental results. The difference is mainly caused by the morphology error during the manufacturing process. The reasons for the error will be analyzed next.

**Table 1:** Experimental, theoretical and optimized simulation diffraction efficiency for the grating.

Polarization	Diffraction order	Results (%)		
		Experimental	Theoretical	Optimized simulation
TE	(−1, −1)	59.6	55.591	94.7
	Total reflection	89.5	97.113	97.1
TM	(−1, −1)	76.7	84.566	93.9
	Total reflection	89.8	96.834	97.9

**Figure 9:**
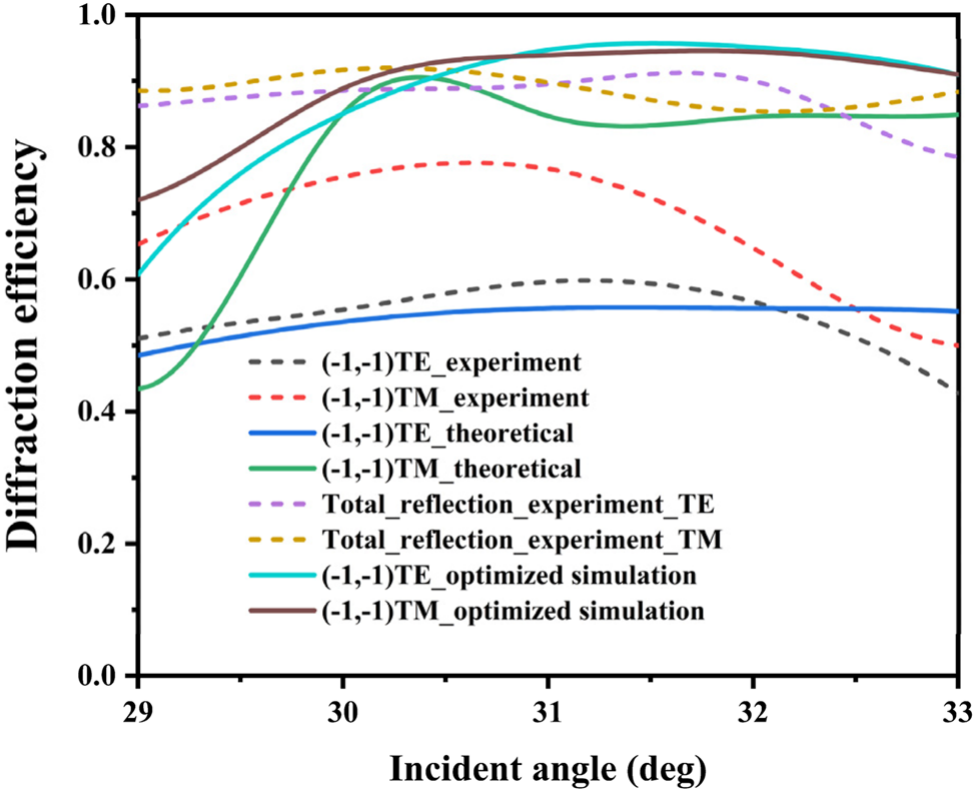
Experiment and optimized simulated efficiency change with angle of incidence.

Although our experimental results verified that the grating achieves both reasonably good efficiency and polarization independent diffraction under 45° azimuth angle auto-collimation condition, the experimental results show non-negligible deviation from optimized results. According to the measured morphology by atomic force microscope (AFM Bruker Dimension 5000), the cylinder height *h*_1_ is 810 nm and the sidewall angle *ψ* is 85.54°, which are different from optimized parameters. Using measured *h*_1_ and *ψ*, the theoretical diffraction efficiencies under TE polarization and TM polarization are 55.951 % and 84.566 %, respectively. As shown in Table 1, the experimental and theoretical results show a deviation of approximately 5 %, confirming the accuracy of the calculation. The deviation mainly comes from the imperfect sidewall angle. Since the height of the grating’s cylinder is larger than its diameter, the inclination of the side wall will tend to be enlarged during development. As shown in Figure 6, the decrease of the sidewall angle will make the high diffraction efficiency region move towards larger duty cycle. When the sidewall angle is 85° and the duty cycle is 0.67, the diffraction efficiency of TM polarization is in the yellow area, while that of TE polarization is in the green area, implying that the diffraction behavior is suppressed. In addition, the proximity effect of the electron beam lithography causes the pattern to be distorted, which also leads to a decrease in diffraction efficiency (Figure 9). The difference between the experimental and theoretical results can also be attributed to photoresist residue. While the simulation assumes a clean grating surface, the fabrication process leaves behind residue and defects during photoresist coating and development, degrading the optical performance of the grating. Simultaneously, Table 1 indicates that material absorption results in a reduction of total reflectance, which will affect the diffraction efficiency, too.

In our work, due to the relatively slow writing speed of electron beam exposure system, it is difficult to manufacture large-sized gratings, which brings uncertainty to evaluate the diffraction efficiency of the grating. Using holographic exposure and reactive ion beam etching can not only achieve frustum structure with better steepness, but also fabricate gratings with larger area. Therefore, choosing the appropriate manufacturing process will further improve the grating performance.

## 6 Conclusions

In summary, we have proposed a two-dimensional polarization-independent grating under auto-collimation configuration with azimuth angle of 45°, of which the cross section is metal-dielectric layer with frustum of a cone structure. When light of 632 nm impinges the grating at the azimuth angle of 45°, the incident angel of 31.6°, the (−1, −1) order will be diffracted back along the incident direction. After optimization of RCWA and SA algorithms, the diffraction efficiency of TE and TM polarization is 95.01 % and 95.04 %, respectively. The electron beam evaporation, vapor chemical deposition and electron beam exposure are carried out to fabricate the final grating pattern to verify the correctness of the simulation. To our knowledge, this is the first time to propose auto-collimation two-dimensional grating when the azimuth angle is 45°. This unique design, production and analysis can be extended to many systems and have potential applications in different fields, such as ultra-fast pulse compression system, spectral combining system and displacement measurement system.
